# On Chorea

**Published:** 1851-01

**Authors:** 


*On Chorea.* By Dr. Lee.—An analysis of various published cases of this disease, and of forty-two observed by himself at the *Hospital des Enfans*, leads Dr. Lee to the conclusion that there are four principal varieties of it.

1. One which has been called *sympathetic*, coincides with the local lesions of the various viscera of organic life, and especially with disease of the gastro-intestinal system and of the heart.

2. A second, which is very common, depends upon a *general* disease, and especially *rheumatism.* So frequent is this variety, that it constituted seventeen out of the author's forty-two cases, and thirty of seventy-four he has collected. Rheumatism indeed may not only give rise to chorea, but to a variety of other nervous disturbances, as simple convulsions, contractions, tetanic convulsions, pseudo-meningitis, pseudo-myelitis, &c.; and, in fact, there is no symptom usually referred to lesions of nervous substance, which may not be dependent upon a rheumatic affection of the joints or heart, such affection being almost always marked by the nervous derangement, and giving rise to only very slight local suffering and febrile action, especially in a chronic neurosis like chorea. When, however, the febrile reaction is intense, the neurosis is usually only developed when the inflammatory fever has undergone some remission; and a reproduction of the febrile action always induces an improvement in the nervous symptoms, except in some cases in which the disease proves quickly fatal.

3. Another form of chorea quite independent of cerebral alteration, is the so-called *essential* chorea, in which no appreciable change of structure is recognisable either in the organic viscera or the nervous system; this, like the rheumatic chorea, is a very common form.

4. The last form depends upon *cerebral* or *spinal lesion,* and is but the symptom of various cerebral and spinal affections.—*Bull de l' Acad.* tom. xv, p. 343.

